# Statistics from One Hundred Patients, Whose First and Second Confinements Occurred under My Observation, Designed to Exhibit the Comparative Duration of First and Second Labors

**Published:** 1869-01

**Authors:** H. W. Dean

**Affiliations:** Rochester, N. Y.


ART. II.—*Statistics from one hundred Patients, whose first and second Confinements occurred under my observation, designed to exhibit the comparative duration of first and second labors.* By H. W. Dean, M. D., *Rochester, N. Y.*

I have adopted in my obstetric records, Murphy's division, dating the commencement of labor with pains recurring at intervals of *half an hour*. The occasional discharge of the *liquor amni* as the first indication of labor, as also the *artificial rupture* of the membranes, sometimes required, cannot be allowed for in statistics of this character, however carefully made up.

Sex of children with primiparæ:

| | |
|---|---|
| Male, | 49 |
| Female, | 51 |
| | 100 |

Average duration of:

| | HS. | MIN. |
|---|---|---|
| 1st stage, | 12 | 53 |
| 2d stage, | 4 | 47 |
| 3d stage, | | 28 |

Average of whole labor, 18 hours, 8 minutes.

Sex with secundiparæ:

| | |
|---|---|
| Male, | 52 |
| Female, | 48 |
| | 100 |

Average:

| | HS. | MIN. |
|---|---|---|
| 1st stage, | 9 | 26 |
| 2d stage, | 3 | 48 |
| 3d stage, | | 25 |

Average of whole labor, 13 hours, 29 minutes, making duration of first labor 4 hours 38 minutes longer than second. By individualizing the cases, however, I find that several patients were much longer in their *second* than *first* confinements.

From a careful analysis of the cases, I find that *sex* had but little influence on the duration of labor, the average with *male* being twenty-three minutes longer than with *female* children. This differs materially from Dr. Collins' report of the Lying-in Hospital of Dublin, who makes the duration with *male* births *one hour and four minutes longer* than with *female.*

Weight seems to have more influence upon the duration of labor than sex. In estimating the primary and secondary labor unitedly, the children weighing over 8 pounds averaged 4 hours and 8 minutes longer in birth than those of less than 8 pounds weight. The weight of *male* children averaged 10 ounces greater than *female.*

Of the 200 children born:

7 presented the breech.
2 presented the knee.
5 presented the feet.
2 presented the face.
3 presented the hand.
12 presented with os frontis to the pubis.

*Duration of Pregnancy:*—Statistics of 77 cases, gathered from the preceding, based upon facts gathered from personal inquiry, and recorded at the time.

Average of whole number 279 days. Three of the cases—respectively, 301, 303, and one 314 days from commencement of last menstrual period. The facts in case of the last mentioned, I transcribe from my note book.

"Mrs. I. R., aged 26 years, healthy and regular in her menstrual periods, (28 and 30 days,) commenced to menstruate on August 29, 1861; was married September 10th, following; never menstruated after marriage; was confined February 12th, 1862, 314 days after last menstrual period."

Abating from the number tabulated—the three cases of over 300 days continuance—the average duration of gestation with the balance was 276 days and a fraction.

